# Low molecular weight heparin in COVID-19: benefits and concerns

**DOI:** 10.3389/fphar.2023.1159363

**Published:** 2023-04-27

**Authors:** Adham Makarem, Rana Zareef, Joseph Abourjeili, Joseph E Nassar, Fadi Bitar, Mariam Arabi

**Affiliations:** ^1^ Department of Health Law, Policy & Management, School of Public Health, Boston University, Boston, MA, United States; ^2^ Faculty of Medicine, American University of Beirut Medical Center, Beirut, Lebanon; ^3^ Department of Pediatrics and Adolescent Medicine, American University of Beirut Medical Center, Beirut, Lebanon; ^4^ Division of Pediatric Cardiology, Department of Pediatrics and Adolescent Medicine, American University of Beirut Medical Center, Beirut, Lebanon

**Keywords:** low molecular weight heparin, lovenox, enoxaparin, COVID-19, SARS-CoV-2

## Abstract

Since its emergence, the COVID-19 pandemic had a dramatic impact on the public health worldwide and it scarred the medical, economical, and social determinants of health. Even after the significant vaccination advances, the disease of SARS-CoV-2 can manifest in severe presentations with life-threatening thromboembolic and multi-organ complications leading to notable morbidity and mortality. Clinicians and researchers are on continuous pursuit of investigating different approaches in the attempt to prevent the infection and minimize its severity. Although the COVID-19’s pathophysiology remains relatively unclear, it is well established now that coagulopathy, systemic thrombotic propensity, and a robust immunoinflammatory response are some of the most important determinants of its morbidity and mortality. Accordingly, research efforts have focused on addressing the inflammatory and hematological cascades using available agents to avoid thromboembolic events. Several studies and investigators have emphasized the importance of Low molecular weight heparin (LMWH), namely, Lovenox, in addressing these sequelae of the COVID-19 disease, either prophylactically or therapeutically. This review explores the benefits and concerns of employing LMWH, a widely used anticoagulant, in COVID-19 disease. It delves into Enoxaparin as a molecule, along with its pharmacology, mechanism of action, and clinical uses. It also reviews the current high-quality clinical evidence that highlight the role of enoxaparin in SARS-CoV-2 infection.

## 1 Introduction

The worldwide public health has been seriously threatened by the coronavirus disease (COVID-19) pandemic. A seafood market in Wuhan City, Hubei Province, China, on December 2019 exposed individuals to the severe acute respiratory syndrome coronavirus 2 (SARS-CoV-2) that was first detected and designated as COVID-19 (Zhu et al., 2020). As it took the world by storm, millions of people have been infected worldwide of whom more than a million patients have succumbed (Moschonas and Tselepis, 2021). Although most SARS-CoV-2 patients exhibited mild to moderate disease, COVID-19 has caused a wide range of more serious illnesses and placed colossal number of patients at greater risk of experiencing morbidity and mortality (Zareef et al., 2022). The most frequent cause of death due to SARS-CoV-2 infection is respiratory failure, but other causes include progression to multiple-organ failure, thrombosis, disseminated intravascular coagulation (DIC), and coagulation activation coupled with excessive immune/inflammatory responses (representing the so-called cytokine storm) (Becker, 2020). Particularly, thrombosis and DIC can cause the patient’s condition to rapidly deteriorate (Asakura and Ogawa, 2021). A rise in plasma D-dimer levels is the most often reported case of COVID-19 related coagulopathy. Elevated D-dimer levels and prognosis have been the subject of numerous research in which elevated D-dimer levels were correlated to poor prognosis (Wu et al., 2020; Zhou et al., 2020).

COVID-19’s pathophysiology remains relatively unclear; however, the pathogenesis of COVID-19 may be significantly influenced by immune-mediated harm where pneumocyte viral infection causes local inflammatory reactions and promotes cytokines release (Shi et al., 2020a). According to accumulating evidence, there are numerous potential cellular and molecular processes that could be responsible for the observed thrombotic consequences. Among them, a pro-coagulant phenotype, platelet activation, and an excessive inflammatory response appear to be key factors in the development of thrombotic problems caused by respiratory virus infections (Moschonas and Tselepis, 2021). In attempts to understand the mechanisms underlying COVID-19-associated coagulopathy and the interplay between the formerly mentioned factors, two theories have been proposed (Yamada and Asakura, 2022). The first theory hypothesizes that when endothelial cells are infected by SARS-CoV-2, they induce thrombosis and vascular inflammation (Yamada and Asakura, 2022). *In situ* hybridization, immunohistochemistry, and electron microscopy have all revealed the presence of SARS-CoV-2 and coronavirus-like particles within the endothelial cells (Yamada and Asakura, 2022). These studies provide credence to the idea that SARS-CoV-2 attacks the endothelial cells directly, impairing their antithrombogenic properties and increase the risk for thrombotic events (Yamada and Asakura, 2022). According to a second theory, thrombosis is not caused by SARS-CoV-2 directly infecting endothelial cells but rather by the cytokine storms brought on by overactive immunological reactions (Yamada and Asakura, 2022). It is important to note that some studies done on endothelial cells *in vitro* demonstrated the resistance of these cells to SARS-CoV-2 infection however, it is the cytokine and chemokine storms, produced by the infected alveolar macrophages and epithelial cells, that caused the endothelial cell damage (Yamada and Asakura, 2022). This damage is brought by platelet activation and coagulation stimulation, which in turn causes an increase in prothrombogenic activity and a decrease in endothelial antithrombogenic activity (Nicosia et al., 2021).

To this end, systemic thrombotic propensity and a robust immunoinflammatory response are characteristics of severe COVID-19. However, it has been suggested that the ‘cytokine storms’ and its consequent thrombotic events should actually be known as “Pulmonary Intravascular Coagulopathy” rather than DIC because the majority of the thrombosis occurs in the lungs as opposed to the entire body involvement seen in DIC (Asakura and Ogawa, 2021). In addition to macro-thrombosis in the lung, this so-called pulmonary intravascular coagulopathy typically manifests more problematic micro-thrombosis across a significant portion of the lung (Asakura and Ogawa, 2021). Subsequently, this necessitates the use of anticoagulant therapy while assuming micro-thrombosis within the lungs, which cannot be seen with contrast-enhanced CT of the chest (Asakura and Ogawa, 2021). Thus, in view of the increasing evidence of thromboembolic events and the noticeable hyperinflammatory state, several studies and investigators have emphasized the importance of Lovenox in addressing these sequelae of the COVID-19 disease, either prophylactically or therapeutically.

In the following review, we investigate the role of Low Molecular Weight Heparin ‘LMWH’ namely, Lovenox, also known as Enoxaparin Sodium, which is a widely used anticoagulant, in COVID-19 disease. We review its pharmacology, mechanism of action, clinical uses, course of treatment pre, post, and during hospitalization, and discuss its potential use for disease prophylaxis *versus* disease therapy. We also highlight the concerns of using this anticoagulant in COVID-19.

## 2 Pharmacology

Enoxaparin, being a low molecular weight heparin (LMWH), has unique pharmacokinetics and pharmacodynamics properties that differ substantially from unfractionated heparin (UFH). Compared to UFH, LMWH has a more favorable side effects profile and more predictable anticoagulant response with better bioavailability, longer half-life, and dose independent clearance (Weitz, 1997). Accordingly, dose adjustments and routine monitoring of anticoagulation activity are not required in most patients (Nutescu et al., 2016). Enoxaparin can be administered either in the subcutaneous forms or via intravenous formulations, but intramuscular administration is generally avoided.

### 2.1 Pharmacokinetically

Enoxaparin is linearly absorbed in the subcutaneous administration and its peak effect (A_max_) is observed after approximately T_max_ = 2.5—4 h (Fareed et al., 2003). When measured in plasma using a standardized amidolytic anti-Xa method, a close relationship between the dose of enoxaparin and anti-Xa A_max_ has been shown to exist. Anti-Factor Xa activity can be detected in plasma for about 12 h after administration (Jupalli and Iqbal, 2022). Moreover, LMWHs have a lower tendency to bind to endothelial cells and thereby are easily absorbed from subcutaneous tissues. Consequently, this enhances the bioavailability for enoxaparin to reach about 91% after subcutaneous administration, which is much higher than the bioavailability of UFH being around 20% (Fareed et al., 2003). Regarding the volume of distribution (Vd) of LMWH, it can be estimated by the anti-Factor-Xa activity. The Vd of enoxaparin is around 5.3 L which is significantly lower than the Vd of other LMWHs. The main reason behind this difference is that enoxaparin exhibits a significantly longer mean residence time (MRT) of around 7 h (Fareed et al., 2003). Enoxaparin is metabolized by the liver via desulfation and/or depolymerization to lower molecular weight species with reduced biologic activity (Nutescu et al., 2016). Concerning excretion, enoxaparin follows first-order kinetics and is eliminated primarily by the kidneys in the urine. Thereby, in contrast to UFH which is dose dependent, the clearance of enoxaparin and other LMWHs does not change as a function of administered dose; they are dose independent (Fareed et al., 2003). This may be attributed to the lower cellular uptake and absorption of enoxaparin when compared with UFH. Following a single dose, the elimination half-life of enoxaparin is approximately 3–4.5 h (Nutescu et al., 2016). In case of repeated doses, its half-life increases to approximately 7 h. The longer elimination half-life of enoxaparin relative to UFH has significant clinical implications, as it reduces the necessity for frequent administration. Worth mentioning, since enoxaparin is primarily eliminated by the kidneys, patients with renal impairment would be at increased risk of drug accumulation and bleeding complications (Fareed et al., 2003; Nutescu et al., 2016; Jupalli and Iqbal, 2022).

### 2.2 Pharmacodynamically

The pharmacodynamic aspect of Enoxaparin is unique as it has enhanced properties compared to unfractionated heparin. They differ markedly in terms of inhibition of coagulation factors, effects on tissue factor pathway inhibitor, platelet interactions, binding to cells and proteins, and other pharmacodynamic properties (Fareed et al., 2003). Enoxaparin has a different degree of inhibition of coagulation factors and a higher ratio of anti-factor Xa to anti-factor IIa activity than does UFH. In addition, both LMWH and UFH lead to the release of tissue factor pathway inhibitor (TFPI), which has inhibitory effects on the coagulation cascade. However, enoxaparin depletes TFPI to a lesser extent than UFH after multiple doses, and consequently, this difference in depletion of TFPI makes the anticoagulant effect of enoxaparin more predictable than that of UFH (Hansen and Sandset, 1998). Moreover, the inhibitory effects of platelet factor 4 (PF4), which is released by activated platelets, is weaker on LMWH than on UFH. Therefore, even in the face of PF4 inhibition, the anti-Xa activity of enoxaparin is still largely intact, and the overall antithrombotic activity is preserved. This reduced interaction with platelets is clinically advantageous for enoxaparin over UFH in that it reduces the incidence of Heparin Induced Thrombocytopenia (HIT) events (Garcia et al., 2012). Also, the lower propensity of enoxaparin to inhibit platelet aggregations will have a decreased tendency to cause bleeding complications. On the second hand, enoxaparin binds to endothelial cells with much lower affinity than UFH which leads to a slower degradation and elimination. This is partly responsible for its increased bioavailability after subcutaneous administration (Fareed et al., 2003). Also, enoxaparin has a reduced propensity to bind nonspecifically to plasma proteins which gives it a clinical advantage over UFH. Plasma protein binding to heparin can reduce the latter’s anticoagulant activity because there will be a lesser concentration available in plasma to interact with antithrombin. Thereby, this can markedly increase the variability in both pharmacokinetic and pharmacodynamic effects (Weitz, 1997). Finally, considering osteoporosis and the side effects of heparin on bones, enoxaparin has a significantly decreased inhibitory effect on alkaline phosphatase activity and little effect on osteoblasts. Consequently, LMWH produce much diluted osteoporotic effects relative to UFH (Fareed et al., 2003).

## 3 Mechanism of action

**FIGURE 1:**
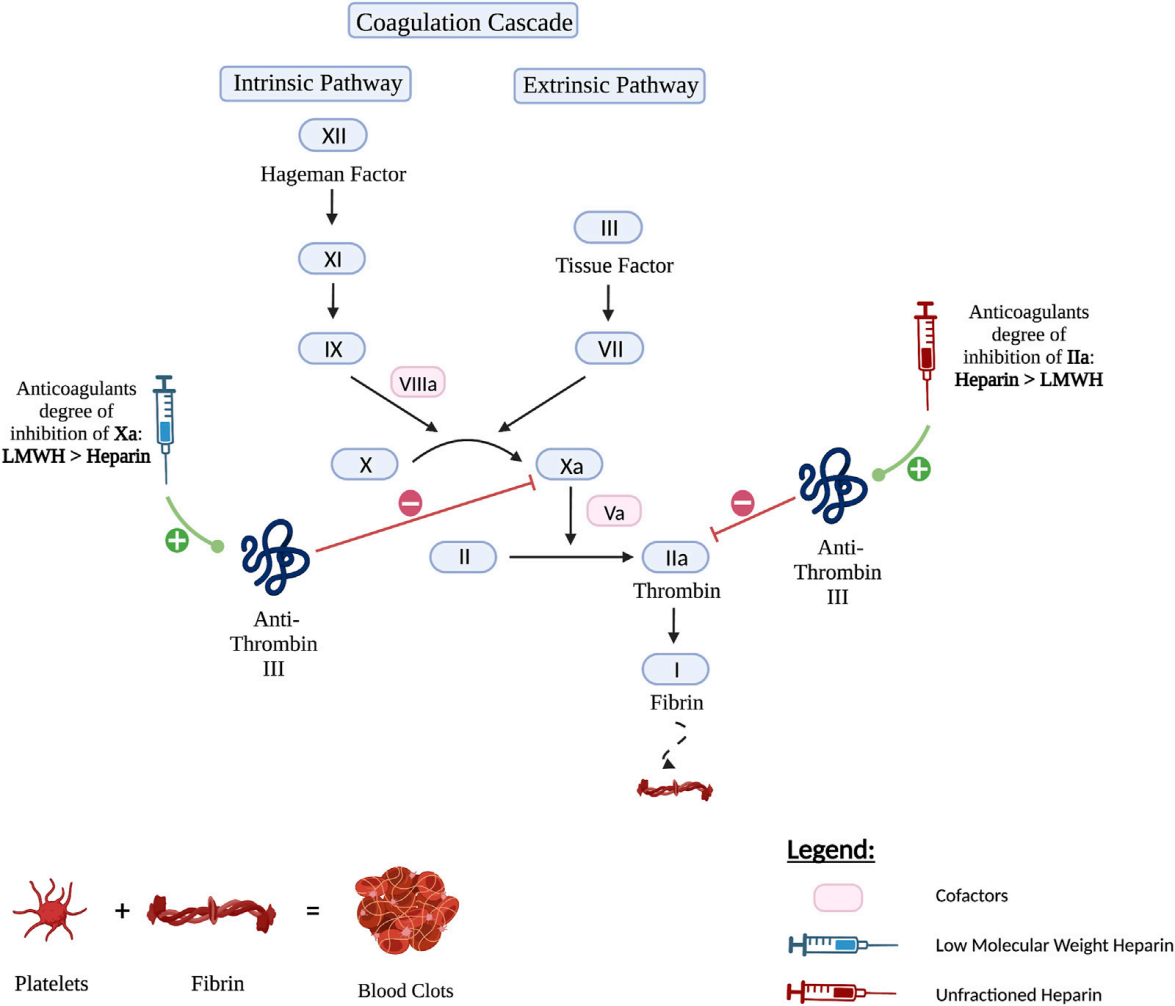
The mechanism of action of LMWH *versus* Unfractionated Heparin. Both Heparin and LMWH possess an anti-coagulant therapeutic effect. They exert their activity by upregulating anti-thrombin III which in turn inhibits factors IIa also known as thrombin and factor Xa. However, Heparin has a higher anticoagulant degree of inhibition of factor IIa than LMWH and therefore inhibits factor IIa more strongly. As for LMWH, it has a higher anticoagulant degree of inhibition of factor Xa and hence inhibits factor Xa more strongly.

Lovenox, or enoxaparin sodium, is a heparin LMWH which is derived by chemical and enzymatic depolymerization of UFH and got approved for medical use in 1993 (Jupalli and Iqbal, 2022). It is a blood thinner that when given in its intravenous form, has a quick onset of action. LMWH is an indirect anticoagulant that exerts its action through binding and potentiating the circulating anticoagulant, antithrombin III (a serine protease inhibitor) through a specific pentasaccharide sequence (Nutescu et al., 2016). Together, they form a complex that irreversibly inactivates factor Xa. Compared to UFH, enoxaparin differs in its relative inhibition of factor II-a, also known as thrombin, and factor Xa. Due to their low molecular weight (4,000–5,000 Da), the small heparin fragments cannot bind thrombin and antithrombin simultaneously. LMWH has lesser degree of inhibition against factor II-a and better activity against factor Xa. Hence, LMWH has decreased activity against thrombin compared to unfractionated heparin. The anti-factor Xa to anti-factor IIa activity ratio for the LMWHs ranges from 2:1 to 4:1, depending on their molecular size distribution (Jupalli and Iqbal, 2022). In contrast, UFH has an anti–factor Xa to anti–factor IIa ratio of 1:1 Important to mention that both LMWH and unfractionated heparin do not break down existing blood clots; they prevent the growth and propagation of formed thrombi (Holbrook et al., 2012). Figure 1 delineates the mechanism of action of LMWH and UFH.

## 4 Clinical uses

LMWH has been in clinical use since 1935 (Qiu et al., 2021). It has largely replaced the use of unfractionated heparin due to its ease of use outside the hospital. In the present time, LMWH is a widely used anticoagulant. It is extensively employed in treatment of venous thromboembolism, cardiovascular disorders, strokes, and thrombosis prophylaxis. LWMH has gradually replaced heparin in the treatment and prophylaxis against thrombosis, since the early 1980s (Qiu et al., 2021). It is a critical therapeutic modality in venous thromboembolism including deep venous thrombosis and pulmonary embolism (Onishi et al., 2016). It is widely used for prophylactic purposes in patients at high risk of venous thromboembolism: those with history of previous venous thrombosis, in cancer patients, and in patients immobilized for long period following a surgical intervention (Onishi et al., 2016). In addition, it is employed in treatment of acute coronary syndrome, with its both sides STEMI and NSTEMI (Howard et al., 2014), as well as acute cerebral infarction (Qiu et al., 2021). Heparin is routinely used in extracorporeal therapy in kidney dialysis and heart-lung oxygenation and as a prophylactic agent when an indwelling catheter is present (Yeo et al., 2015). LMWH is commonly used in pregnancy for treatment and prevention of VTE. Other clinical uses included but not limited to: VTE prophylaxis in nephrotic syndrome, allergic purpura nephritis, recurrent spontaneous abortion, and pre-eclampsia with severe features (Qiu et al., 2021). Beyond anticoagulation, increasing evidence has suggested that heparin exhibits anti-inflammatory properties (Tyrrell et al., 1999).

Heparin is a well-known drug with extensive clinical experience. It is a relatively safe medication; however, it is not free of adverse effects. The major risk is bleeding, which is most prominent in increasing age and in patients with renal impairment. Development of heparin induced thrombocytopenia, osteoporosis, alopecia, and elevation in liver enzymes (Monreal et al., 1989; Paus, 1991; Onishi et al., 2016). Skin necrosis is a very rare yet critical complication with not fully discovered mechanism (Kelly et al., 1981).

## 5 Anticoagulation in COVID-19

**FIGURE 2:**
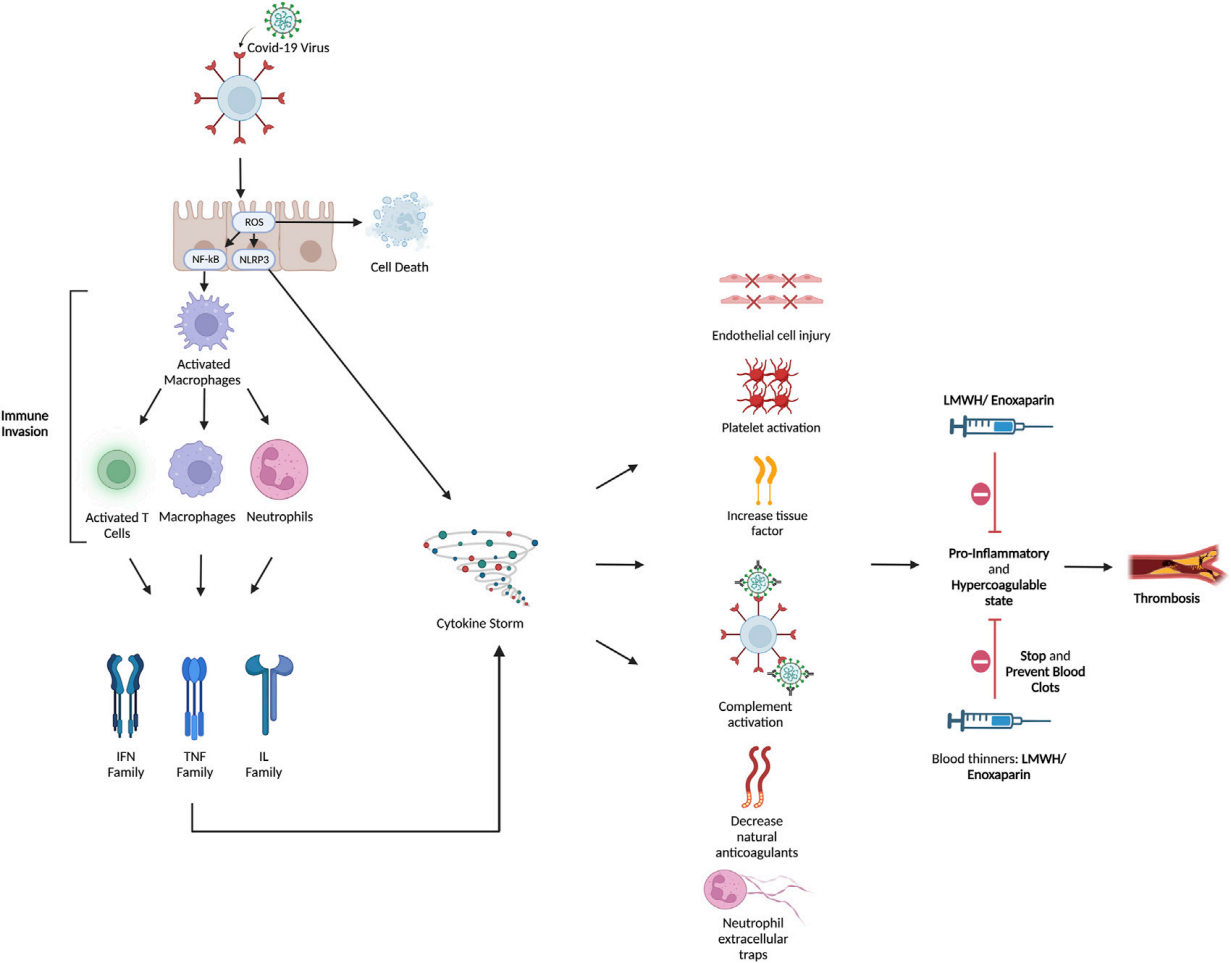
Hyperinflammatory and hypercoagulable conditions were brought on by COVID-19. Although the pathophysiology of the coagulopathy caused by COVID-19 has not yet been fully understood, it has been suggested that immunological dysregulation, hyperinflammation, and thrombosis interact. The SARS-CoV-2 virus enters the cell through endocytosis by interacting with the ACE-2 receptor through its spike protein. Both endothelial and respiratory cells highly express ACE-2. The virus releases its genetic material inside the cell and uses its cellular machinery for replication. There are two proposed methods by which the viral effect occurs: (Zhu et al., 2020) direct viral insult and (Moschonas and Tselepis, 2021) indirect cytokine-mediated injury. The host cell suffers direct harm and undergoes apoptosis as a result of the viral cytopathic impact. In turn, platelet activation and aggregation are brought on by endothelial injury. The virus also causes severe inflammation and immunological dysregulation at the same time. In order to produce pro-inflammatory cytokines including IL-1, IL-2, IL-6, IL-10, IL-17, IL-18, and TNF-, it suppresses lymphocytic activity and activates macrophages and polymorphonuclear cells. This results in a cytokine storm in cases of severe infection. Cytokines and the damaged epithelium cause TF synthesis, VWF release, and triggers the coagulation cascade. The cytokine storm also leads to complement system activation, which causes coagulopathy by causing platelets to become active and boosting the synthesis of fibrin and thrombin. The cytokine storm is linked to NETs, which in turn boost VWF and TF activity and inhibits thrombomodulin and tissue factor inhibitor, leading to inflammation and microvascular thrombosis. Laboratory results are typically notable for a combination of prolonged prothrombin time, normal to mildly prolonged activated partial thromboplastin time, thrombocytopenia, high D-dimer level, fibrinogen, fibrinogen degradation products, VFW, plasminogen, protein C, and factor VIII. Clinically, both the venous and arterial systems are impacted by the hyperinflammatory response and endothelial dysfunction. Here is where heparin and LMWH serve their effect to stop and prevent blot clotting. ACE-2: angiotensin converting enzyme-2; IL: Interleukin; VWF: von Willebrand factor; NET: neutrophil extracellular traps; TF: Tissue factor.

It is strongly evident that COVID-19 infection is associated with hypercoagulability state manifested as microvascular and macrovascular thrombosis and venous thromboembolism (Klok et al., 2020; Li et al., 2020). Unfortunately, the exact etiology of COVID-19 coagulopathy has not been fully discovered. As described above and illustrated in Figure 2 it is suggested to be the result of a combination of endothelial dysfunction, hyperinflammatory state, direct viral injury, and platelet dysfunction (Ackermann et al., 2020). Besides, COVID-19 coagulopathy plays significant role in mortality and morbidity. It is even suggested that higher incidence of thromboembolism occurs in severely ill COVID-19 patients compared to those with mild or moderate infection severity (Cui et al., 2020; Middeldorp et al., 2020; Xu et al., 2020). A positive correlation between incidence of thromboembolism and mortality in COVID-19 has also been investigated (Zhang et al., 2020). Additionally, autopsy studies of COVID-19 patients have revealed wide spread of microvascular thrombosis of pulmonary vessels (Fox et al., 2020), which might contribute to hypoxia and respiratory failure observed in critically ill patients. Therefore, prophylactic, or therapeutic anticoagulation in COVID-19 patients, especially those who are severely affected, is theoretically essential.

Despite the strong evidence of critical thromboembolism in COVID-19, the optimal management of hypercoagulation in COVID-19 patient and the employment of anti-coagulation in their treatment regimen remain unclear. Guidelines and clinical practice rely heavily on the available literature, compromised of observational studies and clinical trials.

Interestingly, at a molecular level, heparin has been suggested to inhibit viral interaction with host cell receptor. In one study, SARS-CoV-2 was found to interact with cellular heparan sulfate (Clausen et al., 2020), making heparin a potent competitive inhibitor for SARS-CoV-2 receptor. In another *in vitro* model, heparin was able to bind to the viral spike protein leading to a conformational change, thus inhibiting cellular entry and invasion (Mycroft-West et al., 2020a). Besides, heparin has shown great capability of decreasing hyperinflammatory state. One study reviewed the medical records of 42 patients with COVID-19 compared D-dimer, fibrinogen, lymphocyte, and IL-6 levels in patients who received LMWH to those who did not. Heparin-treated group had significant decrease in IL-6 level following treatment (Shi et al., 2020b).

Early in the pandemic, studies that assessed the role of anti-coagulation in COVID-19 disease were mostly retrospective observational studies. The initial studies focused on the impact of anticoagulation in decreasing mortality and incidence of thromboembolic events, especially among patients who required intensive care admission. Nevertheless, large variability in choice of anti-coagulation class and dosing were found among the various studies. In addition, the time point at which anticoagulation was initiated is variable among the various studies. Fortunately, around 80% of patients with COVID-19 display mild disease, 15% develop severe disease, while 5% attain a critical state secondary to COVID infection (Turk et al., 2020). It is suggested that the clinical course of COVID-19 follows a 3-phase scenario: the asymptomatic or pre-symptomatic phase, propagating phase, and complicating phase, where each phase is influenced by certain genomics and cellular interactions (Turk et al., 2020). The early phase can be completely asymptomatic or can manifest either with pulmonary symptoms, intestinal symptoms or both. This phase is dominated by the interaction between the SARS-CoV-2 virus and the host ACE-2 receptor, allowing cellular invasion. This is followed by the propagating phase, which constitutes the major determinant of infection severity. It is suggested that during this phase the expression of EGFR and IGFR2R is downregulated, leading to inefficient immune response against the invading pathogen. At this stage, more than two systems are impacted by the virus (Turk et al., 2020). Patients might present with pneumonia, dyspnea, myocardial inflammation, hypercoagulability, or kidney injury. The third phase is the complicating phase is when several systems are heavily affected by the huge viral spread and the clinical status of the patient worsens at this point, severe systemic complications such as ARDS, sepsis, heart failure, coagulopathy are observed (Connors and Levy, 2020; Turk et al., 2020). This phase is impacted by the massive release of cytokines and pro-inflammatory mediators, and the subsequent tissue injury (Hojyo et al., 2020). The hyperactivation of the immune system accompanied by the cytokine storm escort to end-organ damage and mark the last phase of COVID-19 (Hojyo et al., 2020; Turk et al., 2020). In return, the release of cellular contents and endothelial cell injury trigger a hypercoagulable state (Hojyo et al., 2020). Most of the studies examining the role of anti-coagulation in COVID-19 disease focus on their use during the complicating phase and its acute manifestations that include acute lung injury, pulmonary intravascular coagulation, disseminated intravascular coagulation, thrombosis, microangiopathy, myocardial infarction, ischemic strokes, among others (Connors and Levy, 2020). Nevertheless, some studies also tackled the use of anti-coagulation in the early hospital course before displaying severe complications. Below is a discussion of LWMH efficacy in patients with different COVID-19 severities and phases. In addition, table 1 highlights the available high-quality evidence that supports the beneficial impact of LMWH in COVID-19 disease.

**TABLE 1:** The high impact studies tackling anticoagulation in COVID-19. (aHR, adjusted hazard ratio; CI, Confidence interval; AC, anti-coagulation; ICU, intensive care unit; RCT, Randomized Controlled Trial; ECMO, Extracorporeal membrane oxygenation).

Study	Parameters	Site and date	Outcome	Agent, dose, & frequency of LMWH used
Early initiation of prophylactic anticoagulation for prevention of coronavirus disease 2019 mortality in patients admitted to hospital in the United States: cohort study (Rentsch et al., 2021)	Retrospective chart review, 4,297 patients with severe COVID-19 infection	Nationwide study, which includes more than 1,200 points of care in the United States	Decreased mortality (14.3%) in those who received prophylactic anticoagulation compared to those who did not (18%)	*Prophylactic dose:* **Enoxaparin**: SC 40 mg q.d. or 30 mg b.i.d
			The use of prophylactic anticoagulation is associated with 27% decreased risk of 30-day mortality (hazard ratio 0.73, CI 0.66–0.81)	**Fondaparinux**: SC 2.5 mg q.d
				**Dalteparin**: SC 2500–5000IU q.d
				*Therapeutic dose:* **Enoxaparin**: SC > 40 mg q.d
				**Fondaparinux**: SC 5,7.5,10 mg q.d
				**Dalteparin**: SC ≥ 5,500 b.i.d
Trends in venous thromboembolism anticoagulation in patients hospitalized with COVID-19 (Vaughn et al., 2021)	Retrospective study, of 1,351 patients who were hospitalized for COVID-19	30 hospitals across the unites states, March-June 2020	Lower in-hospital mortality among those who received any dose of anticoagulation (prophylactic dose: aHR, 0.36; 95% CI, 0.26–0.52; any treatment dose: aHR, 0.38; 95% CI, 0.25–0.58)	**Enoxaparin (Lovenox):** SC 30–40 mg q.d. or b.i.d
			60-day mortality was decreased with the prophylactic dose of anticoagulation (aHR, 0.71; 95% CI, 0.51–0.90)	**Fondaparinux (Arixtra)**: SC 2.5 mg q.d
Impact of anticoagulation prior to COVID-19 infection: a propensity score–matched cohort study (Tremblay et al., 2020)	Retrospective study of 3,772 hospitalized COVID-19 patients, who either received treatment dose of anticoagulation, prophylactic dose or none during hospitalization	Single center in New York city	Patients on therapeutic dose were more likely to require invasive mechanical ventilation 29.8% vs. 8.1%; *p* < 0.001	LMWH use was not indicated
			Among those on mechanical ventilation, the in-hospital mortality was 29.1% if therapeutic AC was initiated, compared to 62.7% in those who did not receive the therapeutic dose of AC.	
			The risk of mortality decreased with increased duration of therapeutic AC (aHR of 0.86 per day; 95% Cl: 0.82 to 0.89; *p* < 0.001)	
Association of treatment dose anticoagulation with in-hospital survival among hospitalized patients with COVID-19 (Shen et al., 2022)	Retrospective study of 525 COVID-19 patients	2 major centers in China, January-March 2020	Higher survival rate in severely ill patients who received LMWH.	**Enoxaparin:** SC 40 mg q.d or b.i.d
			Those who received LMWH had lower adjusted mortality risk compared to those who did not receive	
Anticoagulant treatment is associated with decreased mortality in severe coronavirus disease 2019 patients with coagulopathy (Tan et al., 2020)	Retrospective, 449 patients with severe covid infection, either received heparin or supportive care	Single center in China, January-February 2020	No difference in 28-day mortality between the two groups	**Enoxaparin:** SC 40–60 mg q.d
			28-day mortality was significantly lower in heparin users among those who had elevated D-dimer level (>6 folds of upper limit) or SIC score of >4	
Heparin in COVID-19 Patients Is Associated with Reduced In-Hospital Mortality: The Multicenter Italian CORIST Study (Di Castelnuovo et al., 2021)	Retrospective observational study, 2,574 hospitalized patients who either received AC or not	Multicenter in 30 clinical centers in Italy, February 2020- June 2020	Patients who received heparin had 40% lower risk of mortality compared to those who received standard of care (HR 0.60; 95% CI: 0.49–0.74)	LMWH agent not specified, dosage used 6,000IU q.d
			The effect was higher with more severe disease	
Therapeutic *versus* prophylactic anticoagulation for severe COVID-19: a randomized phase II clinical trial (HESACOVID). (Lemos et al., 2020)	Randomized controlled open label trial of 20 severely ill COVID-19 patients		Anticoagulation was associated with improvement in oxygenation and higher extubation rate	**Enoxaparin:** SC 40 mg q.d. or 40 mg b.i.d
			No difference in mortality among the two groups	
Efficacy and safety of therapeutic-dose heparin vs. standard prophylactic or intermediate-dose heparins for thromboprophylaxis in high-risk hospitalized patients with COVID-19: the HEP-COVID randomized clinical trial (Spyropoulos et al., 2021)	RCT, 253 patients with COVID-19	12 academic centers across the United States, between May 2020-May 2021	Therapeutic LMWH decreased the incidence of thromboembolic events compared to prophylactic heparin (absolute risk reduction, 13.2%), only in non-ICU patients	*Therapeutic dose:* **Enoxaparin:** SC 1 mg/kg b.i.d. (if CrCl ≥30 mL/min/1.73 m^2^ **)**
			No significant difference in mortality among the different groups	SC 0.5 mg/kg b.i.d. (if 15≤ CrCl<30)
				*Standard dose:* **Enoxaparin** SC 30–40 mg q.d. or b.i.d
				**Dalteparin**: SC 2500IU or 50000IU
Therapeutic Anticoagulation with Heparin in Noncritically Ill Patients with COVID-19 (REMAP-CAP Investigators et al., 2021)	Open-label, adaptive, multiplatform, RCT that included 2,219 non-critically ill COVID-19 patients, who either received therapeutic or prophylactic dose of	Three integrated platforms into a single multiform (ATTACC, ACTIV-4a, REMAP-CAP), 121 sites in nine countries (The United States, Canada, the United Kingdom, Brazil, Mexico, Nepal, Australia, the Netherlands, and Spain), April 2020-January 2021	The probability that Increased organ support–free days at 21 days in the therapeutic group compared to those receiving prophylaxis is 98.6% (aOR 1.27; 95% CI, 1.03–1.58)	Enoxaparin
				Dalteparin
				Tinzaparin
Effectiveness of therapeutic heparin *versus* prophylactic heparin on death, mechanical ventilation, or intensive care unit admission in moderately ill patients with COVID-19 admitted to hospital: RAPID randomized clinical trial (Sholzberg et al., 2021)	RCT, 465 COVID-19 patients with elevated D-dimer, who received either prophylactic or therapeutic	28 sites in Brazil, Canada, Ireland, Saudi Arabia, United Arab Emirates and US. May 2020-April 2021	Reduction in mortality in patients who received therapeutic dose (OR 0.22, CI 0.07–0.65, *p* = 0.006)	*Therapeutic dose:* **Enoxaparin** **^a^** **:** SC 1 mg/kg q12 h or 1.5 mg/kg q24 h or 1 mg/kg q24 h
				**Dalteparin** **^a^** **:** SC 200units/kg q24 h or 100 IU/Kg q12 h or 100 units/kg q12 h
				**Tinzaparin** **^a^** **:** SC 170 U/kg q24 h
				or 175 U/kg q.d
				*Prophylactic dose:* **Enoxaparin** **^a^** **:S**C 40 mg q24 h or 40 mg q12 h
				**Dalteparin** **^a^** **:** SC 5000 units q24 h or 5,000 units q12 h
				**Tinzaparin** **^a^** **:** SC 4500 U or 9,000 ± 1000U q24 h
				**Fondaparinux** **^a^** **:** SC 2.5 mg q24 h
The effect of low-dose and high-dose low-molecular-weight-heparin and aspirin thromboprophylaxis on clinical outcome and mortality in critical ill patients with COVID-19: A retrospective cohort study (Eman et al., 2022)	Retrospective cohort study that includes 164 critically ill ICU COVID-19 patients	Single Center (Sakarya University Hospital, Turkey)	No significant difference in mortality rate or ICU-stay between groups with low-dose LMWH, high-dose LMWH, high-dose LMWH with aspirin	*Low-dose/prophylactic dose:* **Enoxaparin** SC 40–60 mg q.d
		Compromised of COVID-19 ICU patents admitted from March 2020-January 2021		*High dose:* **Enoxaparin** 40–60 mg b.i.d
Intermediate-Dose *versus* Standard-Dose Prophylactic Anticoagulation in Patients with COVID-19 Admitted to the Intensive Care Unit: 90-Day Results from the INSPIRATION Randomized Trial (Bikdeli et al., 2022)	Open-label RCT, 563 ICU COVID-19	Multi-center study, July 2020-November 2020	No difference in outcome in terms of mortality, need for ECMO, and incidence of venous or arterial thromboembolic events among patients receiving standard or intermediate dose of LMWH.	*Intermediate-dose prophylactic:* **Enoxaparin** SC 1 mg/kg q.d
				*Standard- dose prophylactic:* **Enoxaparin** SC 40 mg q.d
Therapeutic Anticoagulation with Heparin in Critically Ill Patients with COVID-19 (The REMAP-CAP et al., 2021)	Open-label RCT, 1,098 critically ill COVID-19 patients	Multi-center over 10 countries: Austria, Brazil, Canada, Ireland, Mexico, Netherland, Nepal, Saudi Arabia, United States, United Kingdom, 2020–2021	Heparin therapeutic dose anticoagulation did not benefit survival or length of organ support in critically ill COVID-19 patients when compared to thromboprophylaxis	LMWH use was not indicated

Abbreviations: aHR, adjusted hazard ratio; CI, confidence interval; aOR, adjusted Odds Ratio; AC, anti-coagulation; q.d., once daily; b.i.d., twice daily; SC, subcutaneous; q24 h, once every 24 h; q12 h, once every 12 h; CrCl, creatinine clearance.

^a^ Doses and frequencies were BMI & CrCl dependent.

### 5.1 Preexisting anticoagulation therapy/pre-hospitalization

The early initiation of anticoagulation has been addressed by several research groups. In a German nationwide study that included 6,637 patients from 854 hospitals, pre-existing anticoagulation use was associated with lower risk of mortality, after adjusting for gender, age and comorbidities (Fröhlich et al., 2021). Chocron et al., assessed the use of anticoagulation before hospitalization on 2878 COVID-19 patients. Authors correlated the use of anticoagulation prior to hospital admission to better outcomes in terms of death and ICU admission. Notably, the two groups did not have similar baseline characteristics. Patients who received pre-admission anticoagulation were more likely to have past medical history significant for diabetes, hypertension, dyslipidemia, or history of previous thromboembolism. However, authors have adjusted for the above parameters (Chocron et al., 2021). In fact, such findings reflects the impact of anticoagulation in the initial phases of infection, specifically during the interaction between the spike protein and the ACE receptor (Mycroft-West et al., 2020b).

### 5.2 Initiation of LMWH during hospitalization

Some studies tackled the impact of anticoagulation initiation during the second and early third phase of COVID-19 illness. They examined the role of anticoagulation in symptomatic disease during viral spread and in those with early complicating phase. A nationwide study in the United States reviewed the charts of patients admitted with COVID-19 to evaluate the impact of prophylactic anticoagulation. Those who received prophylactic anticoagulation within 24 h of hospital admission had decreased risk of mortality (14.3%) compared to those who only received standard of care (18%). Authors found that those who were placed on prophylactic anticoagulation had 27% decreased risk of 30-day mortality (hazard ratio 0.73, CI 0.66–0.81), without increased risk of bleeding (Rentsch et al., 2021). Similarly, the 30-day mortality was lower in patients who received prophylactic or therapeutic anticoagulation during hospitalization in a retrospective study by Vaughn et al., however, the 60-day mortality was decreased in the prophylactic not therapeutic group (Vaughn et al., 2021). Multiple other retrospective studies have agreed on the favorable association between anticoagulation initiation during hospitalization and decreased mortality (Di Castelnuovo et al., 2021; Shen et al., 2022).

### 5.3 LMWH during severe disease

LMWH is also associated with positive impact on complicating phase with severe illness. Among 2,574 patients admitted for COVID-19 infection, the use of LWMH or UFH was associated with 40% decreased risk of in-hospital mortality. Notably, 54% were on therapeutic dose of heparin, and 99.5% of those in the anti-coagulation group were receiving LWMH. The impact was most significant among severely ill patients (Di Castelnuovo et al., 2021). Nevertheless, patients who were placed on anticoagulation had higher prevalence of comorbidities, had more severe illness and were more likely to receive another drug for COVID-19 treatment. After adjusting for these factors, authors have also detected favorable outcomes with heparin use. They later performed subgroup analysis. The positive association of heparin and decreased mortality was more pronounced in patients who had severe illness, who required ICU admission and those who had a D-dimer level greater than 2,020 ng/mL. This finding is also shared by other retrospective cohort analysis (Trinh et al., 2020; Di Castelnuovo et al., 2021; Meizlish et al., 2021; Shen et al., 2022). Indeed, in patients admitted to the ICU and sepsis induced coagulopathy score of >4, thromboprophylaxis was suggested to decrease the mortality rate. This improvement in survival was not observed in patients with score of less than 4 (Tan et al., 2020). Multiple studies have agreed on the association between anticoagulation and decreased rate of mortality without altering the risk of bleeding (Fröhlich et al., 2021; Martinelli et al., 2021; Rentsch et al., 2021; Vaughn et al., 2021), decreased length of hospital stay, and lowered risk of intensive care admission (Albani et al., 2020). For patients who were on mechanical ventilation, the risk of in-hospital mortality was lower in patients who received treatment dose of anti-coagulation compared to prophylactic or no anticoagulation (Trinh et al., 2020; Shen et al., 2022).

### 5.4 Prophylactic vs. therapeutic LMWH

Later, randomized clinical trials were designed and conducted to evaluate the most appropriate dosing. The employment of prophylactic rather than therapeutic does of anticoagulation stems from the increased risk of bleeding observed in COVID-19 patients with high doses of anticoagulation. Indeed, several clinical trials advocate for the use of therapeutic doses. Significant improvement in oxygenation and higher rate of extubating was observed among those who received therapeutic dose of enoxaparin, compared to those who received a prophylactic dose or did not receive anything. Nevertheless, the low number of participants (20 patients) restricts the generalizability of this data (Lemos et al., 2020). Similarly, in the HEP-COVID trial, the use of therapeutic LMWH (Enoxaparin given subcutaneously at 1 mg/kg twice daily or 0.5 mg/kg twice daily) resulted in reduction in the incidence of thromboembolic events without increase in the risk of major bleed (Spyropoulos et al., 2021). The results were only significant in non-ICU patients. The RAPID trial has shown similar decrease in mortality but not in the need for ICU admission or oxygen support (Sholzberg et al., 2021). Notably, this study only included COVID-19 patients with elevated D-dimer level. Martinelli et al., have also compared the outcomes of hospitalized COVID-19 patients who received low dose of LWMH (1 mg/kg once daily) for non-severe cases, intermediate dose (0.7 mg/kg twice daily) for those who have severe illness, and high dose (1 mg/kg twice daily) in ICU patients, in an observational study (Martinelli et al., 2021). The groups had comparable baseline characteristics. Patients on the high-intensity group had lower rates of deaths and incidence of venous thromboembolism. However, a huge limitation in this study is the short study period, where the provided data includes follow-up results for only 21 days (Tan et al., 2020; Martinelli et al., 2021).

Other studies counteract this finding. Data from three randomized trials which were performed in harmony and integrated into a single multiplatform (ATTACC, ACTIV-4a, and REMAP-CAP) showed that the use of therapeutic dose of LMWH has led to fewer days requiring organ support in non-critical patients (REMAP-CAP Investigators et al., 2021). Indeed, no significant advantage was detected of one over the other in altering mortality rate or ICU stay (Eman et al., 2022). Similarly, the use of intermediate dosing (1 mg/kg once daily) *versus* the standard prophylactic dose (40 mg once daily) did not change the mortality rate, need for ECMO, or incidence of venous or arterial thromboembolism within 30 and 90 days of trial initiation (Mazloomzadeh et al., 2021; Bikdeli et al., 2022).

Besides, it is worthy to note that the complications arising from UFH significantly outweigh those of LMWH (Walenga et al., 2004; Al-Eidan, 2015; Monov et al., 2018), thereby making LMWH a better choice for anticoagulation therapy than UFH. For instance, in a retrospective study that analyzed the incidence of HIT among hospitalized patients receiving anticoagulation, the use of UFH was associated with ten times the risk of developing HIT when compared to LMWH (Al-Eidan, 2015). This is primarily attributed to the small molecular size of the LMWH, thus lowering the interaction capacity with PF4 and platelets (Walenga et al., 2004). Congruently, the progression of bone loss at the lumbar spine is found to be accelerated with the long-term use of UFH compared to LMWH (Monov et al., 2018). Similarly, when it comes to COVID-19 infection, one study compared the use of prophylactic and therapeutic LMWH and UFH in management of critically ill 218 COVID-19 patients. LMWH was associated with lower mortality rates (28% vs. 66%), without significant difference in thrombotic or bleeding events (Volteas et al., 2022).

## 6 Concerns

Despite the potential therapeutic advantages of LMWH treatment, and the massive influx of clinical trials examining efficacy and searching for optimal dosing, many studies speculate that LMWH does not have a significant benefit in COVID-19 patients. Table 2 summarizes the high-quality clinical evidence that failed to detect beneficial effect of LMWH in COVID-19 patients. Although such studies are scarce in the literature, they represent a significant finding that would alter the current international treatment guidelines.

**TABLE 2:** The high impact studies tackling the negative outcomes of using LMWH as anticoagulant in COVID-19.

Study	Parameter	Site and date	Outcome of study	Agent, dose, & frequency of LMWH used
Low-molecular-weight heparin compared with Unfractionated heparin in critically ill COVID-19 patients: a meta-analysis (Volteas et al., 2022)	Retrospective cohort study, 218 critically ill intubated COVID-19 patients	Single Center (Stony Brook University Hospital) February-May 2020	No significant mortality benefit with LMWH	**Enoxaparin**: SC 40 mg q.d. (for patients with d-dimer <1,000 ng/mL) Or SC 40 mg b.i.d. (for patients with 1000 ng/mL ≤ d-dimer <3,000 ng/mL) Or SC 1 mg/kg b.i.d. (if d-dimer ≥3,000 ng/mL)
			Increased rate of bleeding and thrombotic complications with the therapeutic use of LMWH compared to UFH	
Thromboprophylactic low-molecular-weight heparin *versus* standard of care in unvaccinated, at-risk outpatients with COVID-19 (ETHIC): an open-label, multicentre, randomised, controlled, phase 3 b trial (Cools et al., 2022)	Open-label RCT, 219 symptomatic COVID-19 outpatients	Multi-center study. It involved 15 centers in six countries (Belgium, Brazil, India, South Africa, Spain, and the United Kingdom), October 2020-November 2021	Early termination of study due to slow recruitment rate, and lower than anticipated events rate	**Enoxaparin:** SC 40 mg q.d. (if weight<100 kg) or 40 mg b.i.d. (if weight ≥100 kg)
			No significant prophylactic benefit of LMWH for symptomatic at-risk patients with COVID-19 in the outpatient setting	
Impact of anticoagulation prior to COVID-19 infection: a propensity score-matched cohort study (Tremblay et al., 2020)	Retrospective chart review of 3772 COVID-19 patients	New York healthcare system, March-April 2020	No significant difference in rate of hospital admission, need for mechanical ventilation or death between groups receiving anticoagulation, antiplatelets or none	LMWH use was not indicated

Among intubated ICU patients with severe COVID-19 infection, the use of anticoagulation did not significantly affect mortality (Volteas et al., 2022). Similarly, the use of low dose, high dose or the addition of aspirin did not alter the mortality and length of stay in patients with severe COVID-19 infection admitted to the intensive care (Eman et al., 2022). Besides, while a study found a positive correlation between heparin and decreased 28-day mortality in severely ill patients with SIC score of greater than or equal 4, it failed to detect a significant impact for LWMH in decreasing mortality in hospitalized patients (Tan et al., 2020). Even in the outpatient setting, a multicenter observational study done in five hospitals in Italy and another high quality randomized controlled trial that was done in 15 centers in six countries could not find a potential therapeutic role for anticoagulation (Russo et al., 2020; Cools et al., 2022). A study was conducted to evaluate patients who had symptomatic COVID infection with at least one risk factor for severe disease (Cools et al., 2022). This study included 219 patients who were randomly assigned to treatment with either enoxaparin (n = 105) or with standard of care (n = 114). After 21 days of treatment, the study was terminated due to slow recruitment rate and lower than estimated velocity of events. Nevertheless, when the obtained data was analyzed, it did not demonstrate any significant difference in outcomes related to mortality or hospitalization between enoxaparin-treated patients and those who were receiving standard care regime (Cools et al., 2022). Therefore, the efficacy and value of LMWH in non-hospitalized and non-critically ill patients must be questioned.

As discussed earlier, the main purpose of employing LMWH in COVID-19 patients is to impede thrombotic events. However, a major complication associated with anticoagulants is a higher incidence of bleeding events (Liu et al., 2020). To further elaborate, LMWH act by blocking the final common pathway in the coagulation cascade: LMWH activates antithrombin III, which consequently binds to factor Xa and inhibits it. This event will inhibit prothrombin conversion into thrombin, consequently inhibiting fibrinogen conversion into fibrin, thereby inhibiting clot formation (Mulloy et al., 2016). Subsequently, by this order of events, there is an increased the risk of bleeding by LMWH due to clot inhibition (Crowther and Warkentin, 2008). Moreover, as emphasized by the above table, several studies conducted on COVID-19 patients receiving LMWH treatment reemphasized the increased incidence of bleeding events induced by LMWH (Bikdeli et al., 2022; Volteas et al., 2022). For instance, In an RCT that showed superiority of therapeutic anticoagulation in increasing organ support-free days, the incidence of bleeding increased from 0.9% to 1.9% in the therapeutic group (REMAP-CAP Investigators et al., 2021). Similar results were observed in other studies (Tan et al., 2020; Shen et al., 2022). Musoke et al. showed that therapeutic anticoagulation is significantly associated with major bleeding events (*p* = 0.04), where 11 out of 102 patients who received therapeutic dose developed major bleeding. They also detected significantly positive association between therapeutic anticoagulation and in hospital mortality (Musoke et al., 2020). In addition, a significant drop in platelet count was noted in 2.2% of patients assigned to the intermediate regimen group (Mazloomzadeh et al., 2021).

Other rare adverse effects pertaining to LMWH are osteoporosis, heparin induced thrombocytopenia (HIT), and hypoaldosteronism (Bengalorkar et al., 2011; Yesasuri and Honasoge, 2018). It is also important to assess for potential LMWH induced hyperkalemia. LMWH induces hypoaldosteronism by decreasing the total number and affinity of angiotensin II receptors. This will subsequently leads to hyperkalemia which can cause life threatening arrythmias (Simon et al., 2021).

## 7 Conclusion

The COVID-19 pandemic has posed significant burdens on the global health and economic sectors, leaving behind substantial morbidity and mortality. While the search for a therapeutic agent is still ongoing, the scientific community succeeded in obtaining the vaccine which limited the drastic consequences and widespread of the virus. Meanwhile, despite the strong evidence of critical thromboembolism in COVID-19 infection and its association with increased morbidity and mortality, the optimal management of hypercoagulation in COVID-19 patient and the employment of anti-coagulation in their treatment regimen remain unclear. In this review paper, we investigated the role of LMWH as an effective anticoagulant in COVID-19 disease. Although LMWH has shown to have a marked capability of decreasing hyperinflammatory state and IL-6 levels, multiple studies displayed different therapeutic and prophylactic effects of LMWH in COVID-19 patients. Other articles highlighted the many concerns and side effects of Lovenox in this particular population. Accordingly, more research studies, including multi-center placebo-controlled high-quality randomized clinical trials with plainly outlined baseline characteristics and outcomes, are urgently needed to evaluate the efficacy of LMWH in COVID-19 disease and to better define recommendations for clinical practice.
